# Conversion of UKA to TKA using identical standard implants—How does it compare to primary UKA, primary TKA and revision TKA?

**DOI:** 10.1186/s42836-024-00267-x

**Published:** 2024-09-03

**Authors:** Christian B. Scheele, Matthias F. Pietschmann, Thomas C. Wagner, Peter E. Müller

**Affiliations:** 1 Department of Orthopedics and Trauma Surgery, Musculoskeletal University Center Munich (MUM), University Hospital, Großhadern Campus, Ludwig Maximilians University, Marchioninistr. 15, Munich, 81377 Germany; 2 Department of Orthopedics and Sports Orthopedics, Klinikum Rechts Der Isar, Technical University Munich, Ismaninger Str. 22, Munich, 81675 Germany

**Keywords:** Functional results, Patient satisfaction, Revision arthroplasty, TKA, UKA

## Abstract

**Background:**

UKA is a well-established treatment option for anteromedial osteoarthritis of the knee, resulting in superior functional outcomes but also higher revision rates than TKA. This study aimed to compare the outcomes of UKA, TKA, UKA converted to TKA using identical standard implants and revised TKA to support clinical decision-making.

**Methods:**

In this study, we retrospectively examined 116 patients who underwent UKA, 77 patients who received TKA, 28 patients whose UKA was converted to TKA using identical standard implants, and 21 patients who had a one-stage revision of TKA. The mean age at operation was 66.5 years (39–90 years), with a mean BMI of 28.8 kg/m^2^ (17.4–58.8) and a mean follow-up period of four years (0.9–9.9 years). We assessed various PROMs, including Oxford Knee Score, UCLA score, KSS score, and a modified WOMAC-Score as well as patient satisfaction and ability to resume daily activities, work, and sports.

**Results:**

The highest patient satisfaction was seen in the UKA. All scores were significantly higher for UKA than for TKA, converted UKA, and revised TKA. None of the scores showed a significant inferiority of converted UKA to TKA. In the case of revision, two scores showed significantly better results for converted UKA than for revised TKA.

**Conclusions:**

Our results indicated that patients initially treated with UKA did not have significantly worse functional outcomes after conversion to TKA, given the use of identical standard implants. This highlights the effectiveness of UKA as a therapeutic option with outcomes superior to those of primary TKA and the importance of a bone-sparing procedure. Conversely, revision TKA is linked to poorer functional outcomes compared to both primary arthroplasties.

## Introduction

For anteriomedial osteoarthritis of the knee, unicompartmental knee arthroplasty (UKA) is an excellent treatment option, with better functional outcomes and fewer complications compared to total knee arthroplasty (TKA) [1–3]. However, the lifetime risk of revision for UKA is twice that of TKA across all age groups, ranging from 3.7% to 40.4% for UKA and 1.6% to 22.4% for TKA [4]. Aseptic loosening, pain and disease progression have been shown to be the main reasons for UKA revision [5]. It is noteworthy that periprosthetic joint infections accounted for only 4% of all UKA revisions, compared to 27% for TKA, which allows for a one-stage revision in almost all cases [4, 5]. When UKA revision becomes necessary, the general recommendation is to convert to TKA [6].

Probably because of this difference in revision rates, although 30%–50% of knee osteoarthritis patients are eligible for UKA, most ultimately choose TKA [7–9]. While several studies have reported the relative ease of UKA revision over TKA revision, which is sometimes even considered to be a factor driving UKA revision rates [3, 6, 10], the most fundamental aspect of the trade-off between the superior functional results of UKA and the lower revision rates of TKA is the clinical outcome of UKA converted to TKA versus primary TKA. There is an ongoing debate, with some studies supporting that revising failed UKA achieves comparable outcomes to primary TKA [11–13], while others claim the opposite [14–16]. A confounding factor in previous studies may be that the implant types in the UKA-to-TKA conversion group were very heterogeneous [11, 12, 15, 17–19], with two meta-analyses showing that UKA-to-TKA conversion is much more likely to require revision components, or at least a thicker polyethylene component, than primary TKA [20, 21].

The aim of this study was to compare patient satisfaction, physical activity data and knee-specific functional outcomes of primary UKA, primary TKA, converted UKA and revised TKA to support clinical decision-making and to optimize treatment strategies for patients who are candidates for UKA. To the best of our knowledge, this was the first study to analyze identical implants in both the revision UKA and the primary TKA groups.

## Methods

**Fig. 1:**
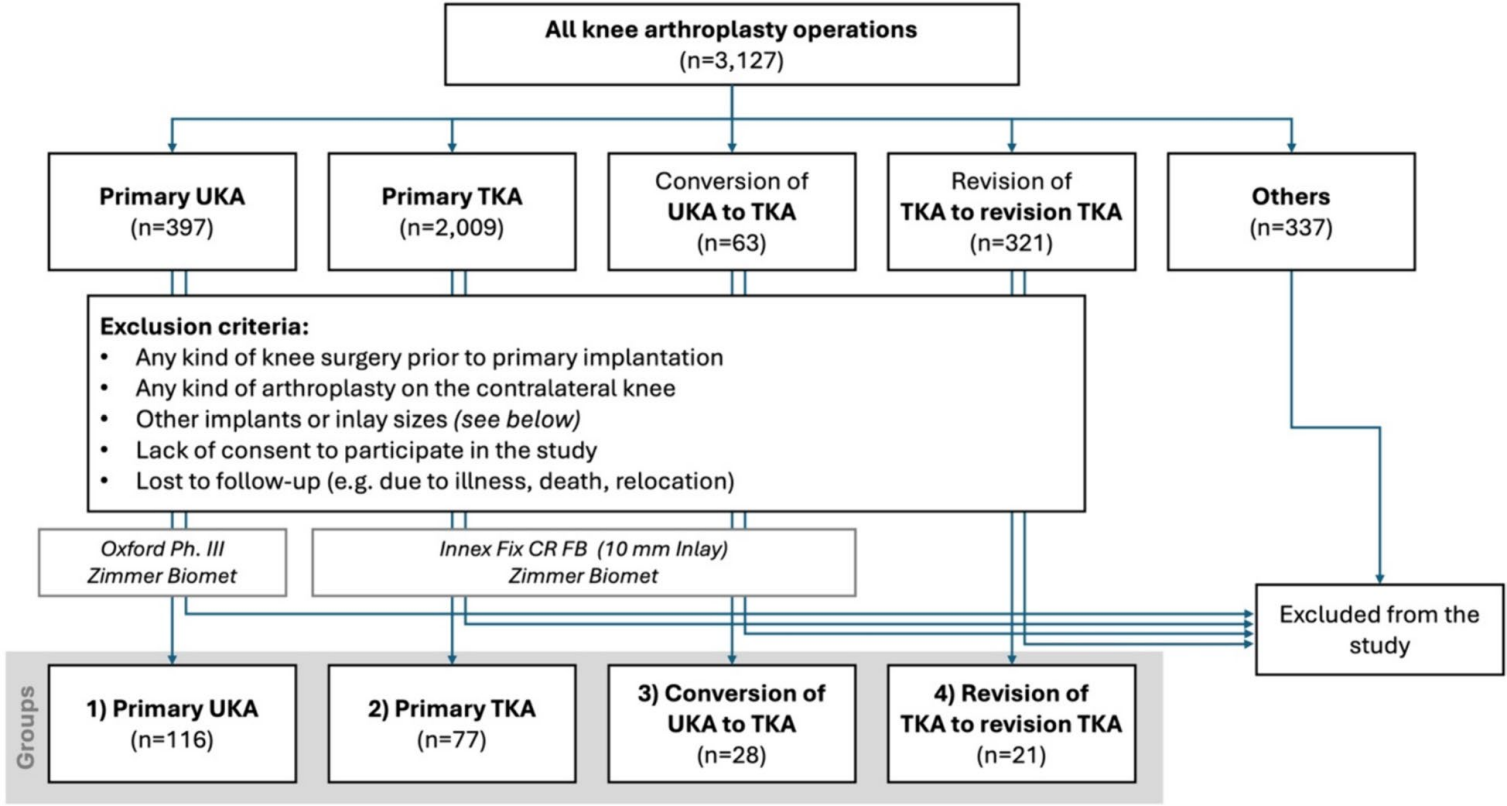
Flow chart showing the group distribution and exclusion criteria (period under review from 1998 to 2011)

The study concept received approval by the local Ethics Committee and all patients signed written consent before being included into the study. All arthroplasties assessed in this study were performed between 1998 and 2011. The following four groups were analyzed: (1) Primary UKA, (2) primary TKA, (3) conversion of failed UKA to TKA and (4) revision of failed TKA to revision TKA. The operative procedure as well as the postoperative treatment were similar in all patients. Implants used in this study were cemented Biomet Oxford Phase III in the UKA group and cemented Innex Fix CR Fixed Bearing Inlay with 10 mm Inlay (Zimmer Biomet, Warsaw, USA) in both the primary TKA and the converted UKA groups. Patients who received any kind of knee surgery prior to primary implantation or an arthroplasty on the contralateral knee were excluded from the study. In addition, patients treated with other implants or other inlay sizes were also excluded from further examination (Fig. 1).

The patients’ characteristics including age, gender, operated side, body mass index (BMI) and follow-up period were recorded from the medical case records.

The UKA group included 116 patients (65 female, 51 males; 52 right, 64 left). The average age at the time of surgery was 65.9 ± 8.4 (43.6–90.2) years. Follow-up lasted 4.2 ± 2.6 (0.9–9.9) years after implantation.

The TKA group consisted of 77 patients (49 female, 28 males; 54 right, 23 left). The average age at implantation was 67.2 ± 8.6 (38.8–82.0) years. Follow-up examinations were performed, on average, 4.0 ± 1.7 (2.3–8.6) years postoperatively.

As with TKA, the converted UKA group only included patients who received a Zimmer Biomet Innex Fix CR Fixed Bearing with 10-mm inlay height. There was no need for stems or wedges. However, in some cases we transferred an autologous bone slice from the lateral to the medial proximal tibia to restore bone stock. All patients received both primary implantation of the UKA and conversion to TKA at our institution, ensuring that surgical technique and postoperative care were comparable. 28 patients were included (17 female, 11 males; 15 right, 13 left). The age at primary implantation was 61.7 ± 8.5 (42.8–75.3) years, the age at revision from UKA to TKA was 64.9 ± 9.0 (46.0–77.2) years. The follow-up period after conversion was 3.2 ± 1.9 (0.4–8.7) years.

The group of revised TKA comprised 21 patients (14 female, 7 males; 12 right, 9 left). The average age at primary implantation was 66.8 ± 7.6 years, at revision 69.1 ± 7.5 years (average lifetime of prosthesis 2.3 ± 1.6 years). The follow-up period lasted for 5.7 ± 1.9 years after primary implantation and 3.4 ± 1.5 years after revision surgery.

The functional outcome of the patients was assessed using a set of questionnaires consisting of 91 self-reporting questions. Of these, 39 questions related to the pre- and postoperative condition, and 13 further general questions to the current state of health, possibly pre-existing conditions, previous surgeries, or the occupation performed. Each patient answered (1) the Oxford Knee Score [22], (2) the UCLA-Score (University of California, Los Angeles activity scale) [23], (3) the KSS (Knee Society Score) [24] and (4) the WOMAC-Score (Western Ontario and McMaster Universities Osteoarthritis Index) [25, 26].

Concerning the WOMAC-Score, in contrast to the original score, we used 11 possible answers per question (from “I feel no pain” [0 points] to “I feel very strong pain” [10 points]) and calculated and indicated the reciprocal value of the determined scale values as well as for the total score, so that, in contrast to the original score, we had a good functionality of the joint with high results.

In addition to the questionnaires answered, all patients were invited for a detailed clinical follow-up examination, which was performed 6 months postoperatively at the earliest so that potential operation-associated conditions did not alter clinical outcomes.

The statistical evaluation was carried out using Graph Pad Prism 6 and Microsoft Excel 2010. For the comparative calculation of the results in the various groups, we used the Kruskal-Wallis-Test. A Dunn-Bonferroni test was employed to pairwise compare the groups to determine which were significantly different. The Mann-Whitney U test was utilized to compare two groups. The significance level was set at a *P* < 0.05.

## Results

There were no significant differences in age, BMI or sex distribution at the time of implantation (Table 1). On average, follow-up took place 4.0 years postoperatively, with no significant differences between the groups.

**Table 1:** Patient characteristics

	**UKA**	**TKA**	**Converted UKA**	**Revised TKA**	***P*-value**
**Patients**	*n* = 116	*n* = 77	*n* = 28	*n* = 21	
**Age at operation (years)**	65.9 SD 8.4 (43.6–90.2)	67.2 SD 8.6 (38.8–82.0)	64.9 SD 8.9 (46.0–77.2)	69.1 SD 7.5 (46.6–79.9)	*P* = 0.229
**Sex (m/f)**	51/65 (44.0% vs. 56.0%)	28/49 (36.4% vs. 63.3%)	11/17 (39.3% vs. 60.7%)	7/14 (33.3% vs. 66.7%)	*P* = 0.665
**BMI (kg/m**^**2**^**)**	28.7 SD 4.8 (19.1–56.1)	28.4 SD 5.2 (20.2–45.3)	29.2 SD 4.7 (21.6–39.8)	29.9 SD 8.7 (17.4–58.8)	*P* = 0.697
**Follow-up examination**	4.2 SD 2.6 (0.9–9.9)	4.0 SD 1.7 (2.3–8.6)	3.2 SD 1.9 (1.0–8.7)	3.4 SD 1.5 (1.4–6.2)	*P* = 0.110
**Implant**	Biomet Oxford Phase III	Innex Fix CR Fixed Bearing	Innex Fix CR Fixed Bearing		

**Fig. 2:**
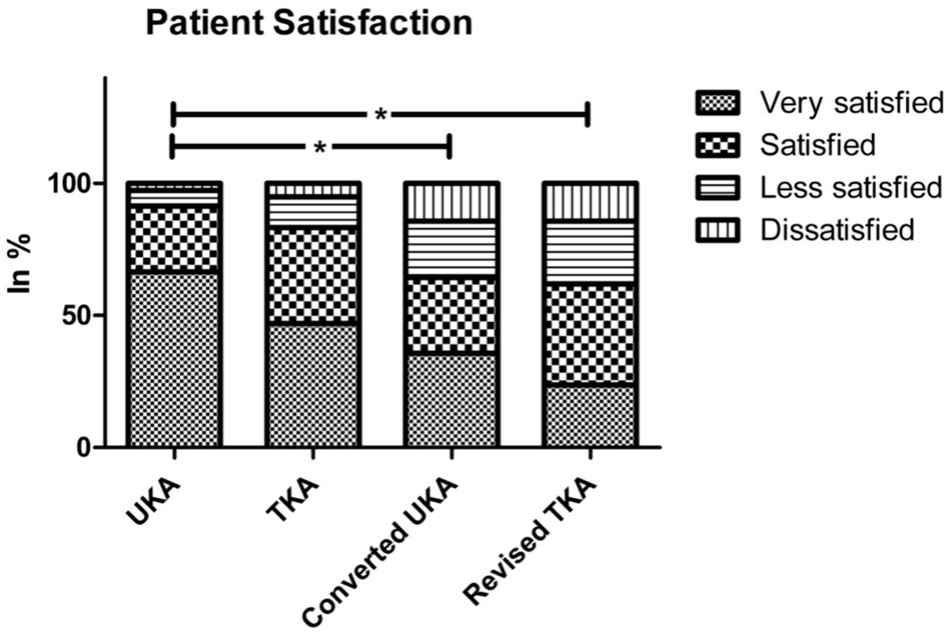
Patient satisfaction: significant inferiority of revision arthroplasty only compared to UKA. No significant difference in patient satisfaction comparing UKA and TKA (*P* = 0.054); * = significant difference (*P* < 0.05)

Overall, 66.4% of patients in the UKA group, 46.8% of patients in the TKA group, 35.7% of patients in the converted UKA group, and 23.8% of patients in the revised TKA group were very satisfied with the postoperative result (*P* < 0.001; Fig. 2). While primary UKA was significantly superior to converted UKA and revised TKA, there were no significant differences between UKA and TKA (*P* = 0.054) or between primary TKA and converted UKA (*P* = 0.662). After primary arthroplasty, patient satisfaction was, on average, higher in males than in females, without reaching the level of significance (*P* = 0.223). In the group of UKA patients, 49 out of 51 men (96.1%) said they were satisfied or very satisfied. Among women, 57 of 65 (87.7%) were satisfied or very satisfied (*P* = 0.442). With regard to primary TKA, 25 out of 28 men (89.3%) and 38 out of 49 women (77.6%) were satisfied or very satisfied (*P* = 0.398).

**Fig. 3:**
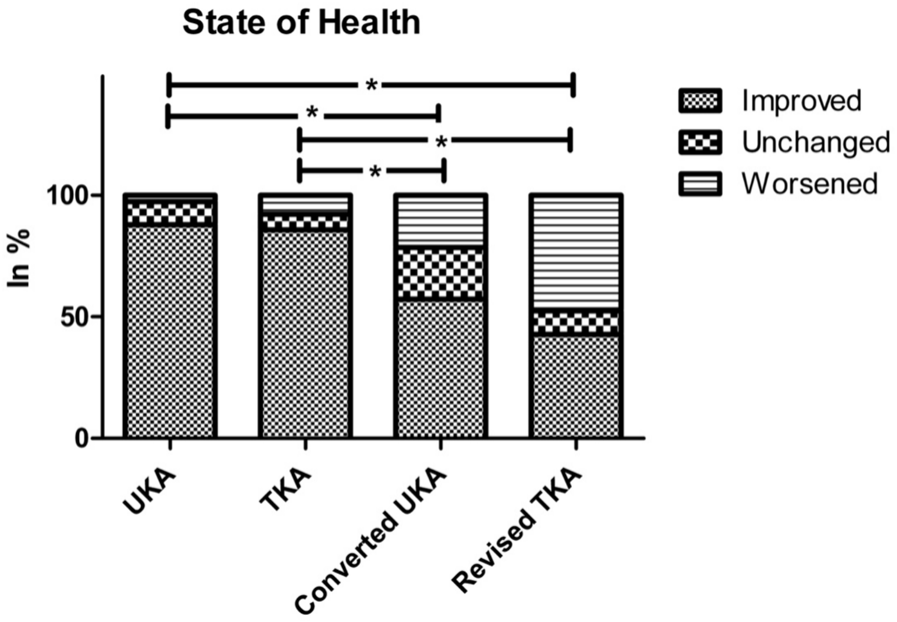
Subjective perception of own health status is significantly worse after revision arthroplasty than after primary arthroplasty; * = significant difference (*P* < 0.05)

The postoperative overall state of health was rated as improved by 87.9% of UKA patients (stable: 9.5%; worsened: 2.6%), 85.7% of TKA patients (stable 6.5%; worsened: 7.8%), 57.1% of converted UKA patients (stable: 21.5%; worsened: 21.4%) and 42.9% of revised TKA patients (stable 9.5%; worsened: 47.6%). The postoperative overall health status was significantly lower after revision surgery than after primary implantation (*P* < 0.001; Fig. 3).

**Fig. 4:**
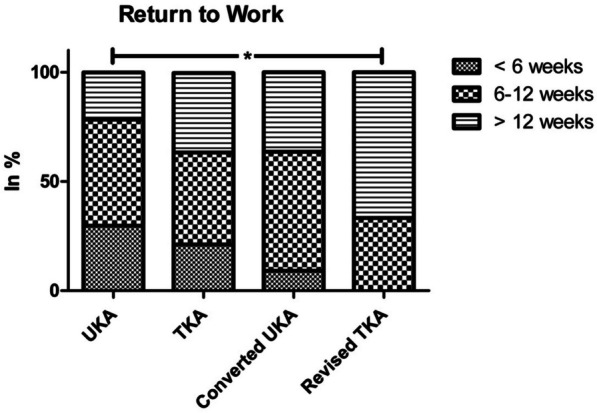
Time required to return to work. Significant differences only in comparison of primary UKA and revision TKA; * = significant difference (*P* < 0.05)

**Fig. 5:**
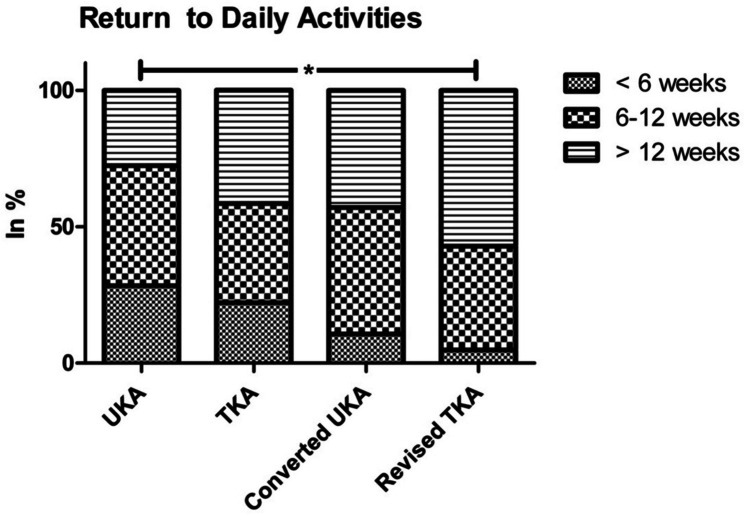
Time to resumption of daily activities by study groups. Significant differences only in comparison of primary UKA and revision TKA; * = significant difference (*P* < 0.05)

On average, the UKA group had the shortest time required to return to work and activities of daily living and was followed by TKA patients. In approximately a similar proportion of TKA and converted UKA patients (36.8% vs. 36.4%), more than 12 weeks were required. Patients with revised TKA required significantly more time than those with primary UKA (*P*_work_ = 0.018; *P*_daily activities_ = 0.008; Figs. 4 and 5). The ability to do sports improved in 53.4% of UKA patients, 33.3% of TKA patients, 32.1% of converted UKA patients, and 15.8% of revised TKA patients, with only the difference between UKA and revised TKA being significant (*P* = 0.001).

**Fig. 6:**
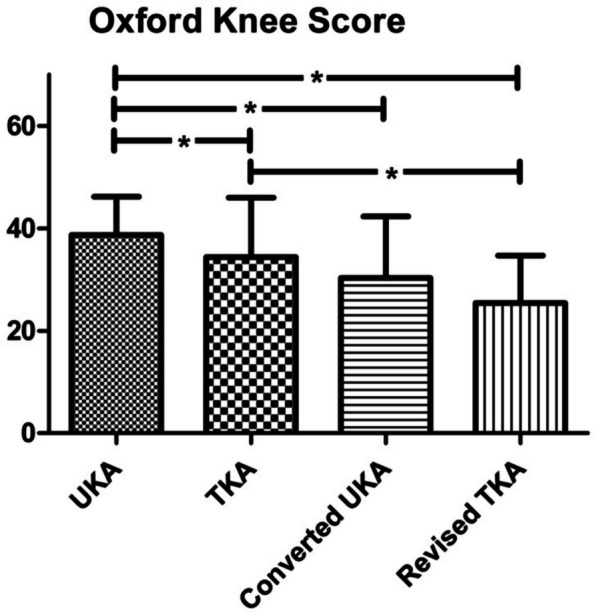
Oxford Knee Score: UKA shows significantly superior results compared to all other groups. Revision of TKA shows significant inferiority to primary TKA. No significant difference between primary TKA and UKA converted to TKA.; * = significant difference (*P* < 0.05)

The Oxford Knee Score was significantly higher for primary UKA (38.7 SD 7.5) than for primary TKA (34.4 SD 11.6), converted UKA (30.3 SD 12.0) and revised TKA (25.4 SD 9.3; *P* < 0.001). In contrast, the difference between TKA and converted UKA as well as converted UKA and revised TKA did not reach the level of significance. Revised TKA led to significantly inferior results than primary TKA (Fig. 6).

**Fig. 7:**
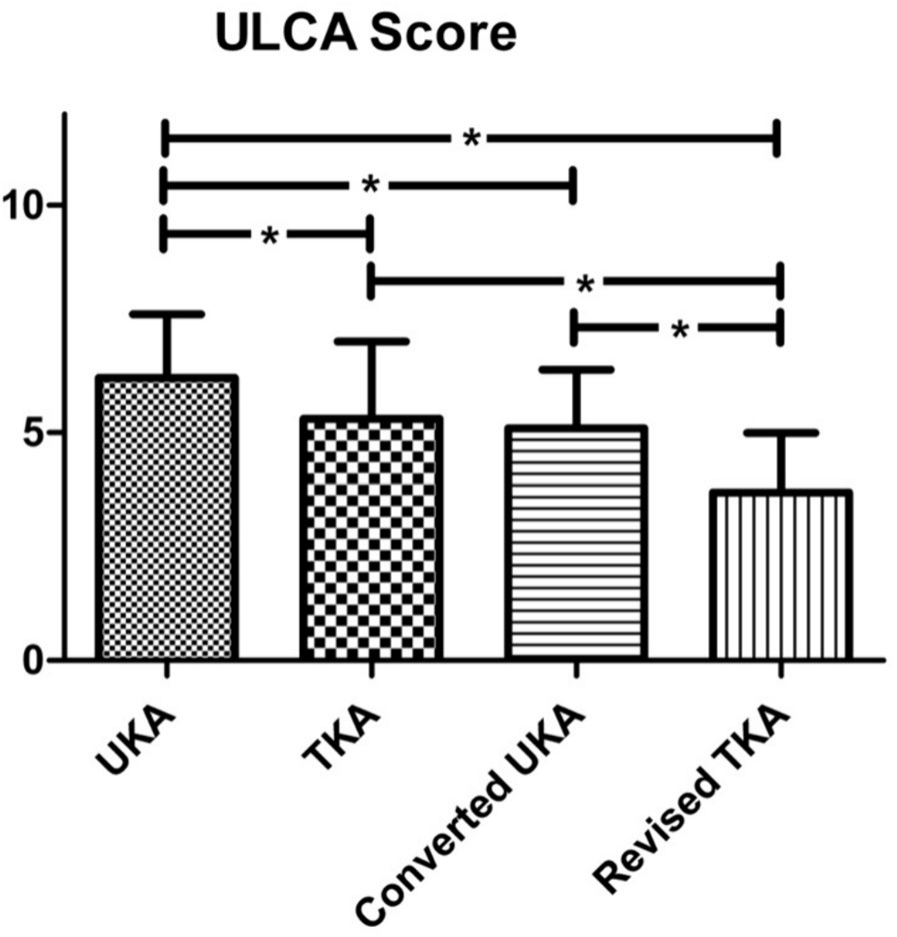
ULCA score: UKA shows significantly better results than all other groups. No significant difference between primary TKA and UKA converted to TKA. Revision of TKA shows significant inferiority to primary TKA.; * = significant difference (*P* < 0.05)

The functional outcome in terms of the UCLA Score was significantly better for primary UKA (6.2 SD 1.4) than for primary TKA (5.3 SD 1.7), converted UKA (5.1 SD 1.3) and revised TKA (3.7 SD 1.3; *P* < 0.001). Converted UKA were not inferior to primary TKA but significantly superior to revised TKA. Revised TKA led to significantly worse results than primary TKA (Fig. 7).

**Fig. 8:**
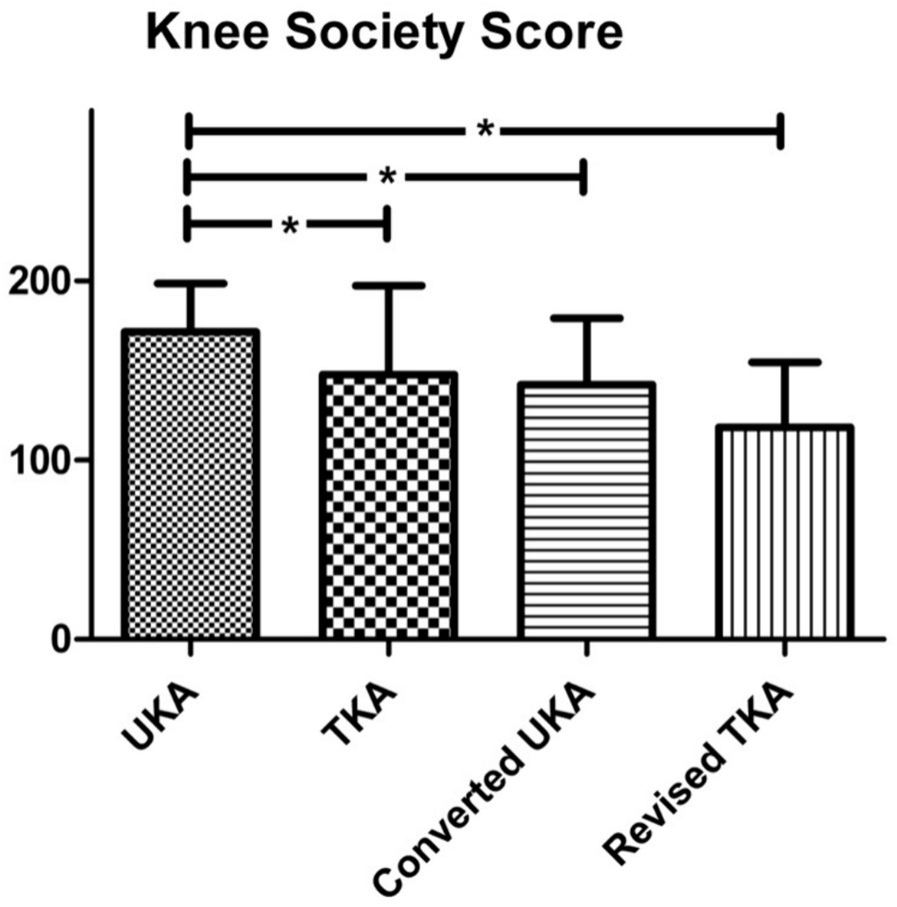
Knee Society Score (KSS): UKA showed significantly superior results in KSS than all other groups. No significant difference between primary TKA and UKA converted to TKA; * = significant difference (*P* < 0.05)

The KSS showed significantly higher scores for primary UKA (overall: 171.4 SD 27.2) than for primary TKA (overall: 147.7 SD 49.6), converted UKA (overall: 142.0 SD 37.0) and the revised TKA (overall: 118.0 SD 36.5; *P* < 0.001). Furthermore, there was no significant difference between converted UKA and primary TKA. Converted UKA was significantly superior to revised TKA in terms of the functional score (64.4 vs. 46.5), but not in terms of the knee (77.6 vs. 71.5) or overall score (142.0 vs. 118.0). Revised TKA was rated significantly worse than primary TKA in terms of the functional score (46.5 vs. 73.0) but not in terms of the knee (71.5 vs. 74.7) or overall score (118.0 vs. 147.7; Fig. 8).

**Fig. 9:**
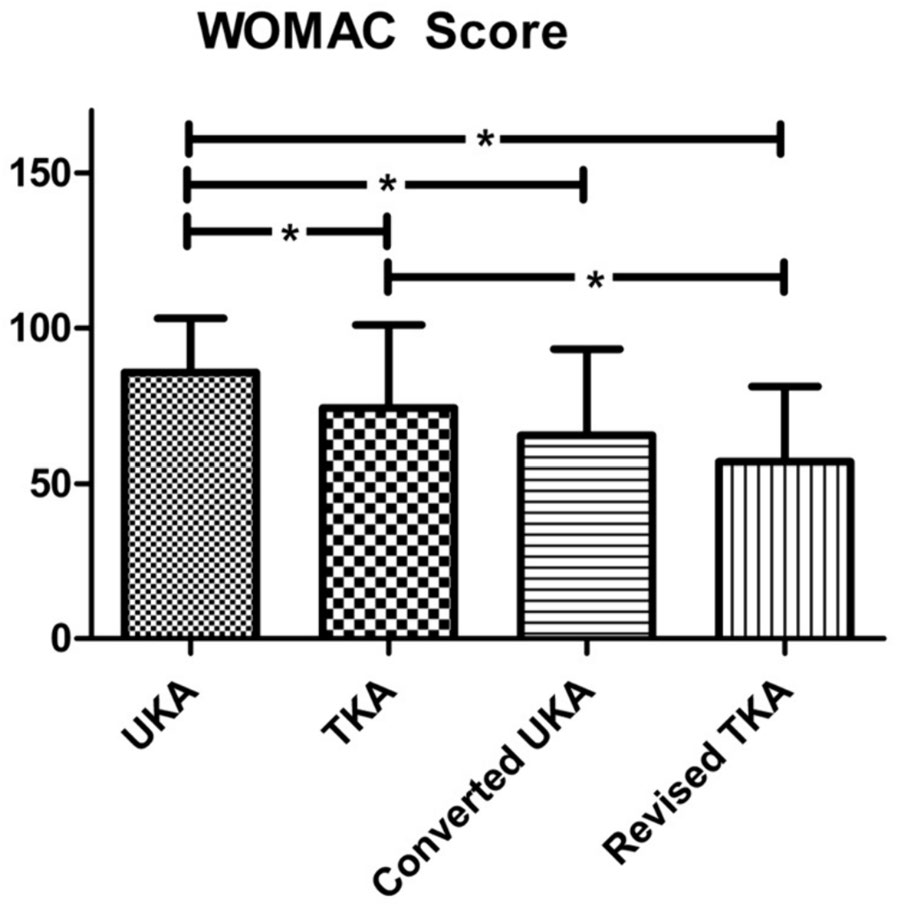
Modified WOMAC score: UKA shows significantly better results than all other groups. No significant difference between primary TKA and UKA converted to TKA. Revision of TKA shows significant inferiority to primary TKA; * = significant difference (*P* < 0.05)

Regarding the modified WOMAC Score, primary UKA was rated significantly superior to primary TKA in the overall (85.7 vs. 74.2), pain (88.3 vs. 76.4) and functional section (86.3 vs. 73.6). The difference concerning stiffness did not reach the level of significance. Primary UKA performed significantly better than converted UKA and revised TKA in all subsections. Primary TKA was superior to revised TKA in the overall perspective (74.2 vs. 56.9) and with respect to the functional results (73.6 vs. 55.4). However, primary TKA was not significantly better than converted UKA, neither in any subsection nor in the overall perspective (Fig. 9).

The proportion of patients with extension lag did not differ significantly between the groups (*P* = 0.9038). The average flexion ability was significantly higher in the UKA group than in any other group and significantly higher in the TKA group than in the revised TKA group (*P* < 0.0001).

## Discussion

Functional outcomes following the revision of UKA to TKA relative to primary TKA have been a topic of interest in orthopedic research. While a meta-analysis of matched studies by Levy et al. [27] reported comparable outcomes for UKA revised to TKA and primary TKA, a number of studies reported inferior functional outcomes and higher complication rates for revised UKA [11, 15, 17, 18]. In particular, two meta-analyses conducted by Zuo et al. (2018) and Sun and Su (2018) found that conversion of UKA to TKA was associated with worse functional outcomes than primary TKA, but no significant difference was found in terms of postoperative complications, blood loss and length of hospital stay [21, 28]. However, these studies could not explain the underlying mechanism of the observation that converted UKA had inferior outcomes compared to TKA. In the meta-analysis by Sun and Su [21], the need for augmentation, stems and bone grafts was 100 times higher in the converted UKA group (37.0%) than in the primary TKA group (0.3%), and the mean polyethylene thickness was significantly greater in the converted UKA group (12.4–13.9 mm) than in the primary TKA group (10.3–11.2 mm). As heterogeneous implant types, also observed in a review by Lee et al. [20], in the UKA revision group could alter functional outcomes compared to primary TKA, we decided to compare the functional outcomes of primary UKA and primary TKA with a best-case scenario of UKA revision, including only UKA revisions using identical components as in the primary TKA group. As Lunebourg et al. [29] found that functional scores and quality of life after converted UKA were more comparable to revision TKA than primary TKA, a group of TKA revisions was also included in the study.

Regarding the four knee scoring systems, primary UKA had the best results and was followed by primary TKA, converted UKA, and revised TKA. Primary UKA was significantly superior to primary TKA in all assessed scores, while there were no significant advantages of primary TKA over converted UKA. Therefore, it appears that patients treated with UKA do not have to fear significantly worse results after conversion to TKA compared to patients initially treated with TKA, provided that UKA revisions involving stems, augments, and different polyethylene components can be avoided. We suggest that a bone-sparing UKA implantation could facilitate the use of standard implants in revision scenarios, leading to long-term success, particularly in young patients who have the highest lifetime risk of revision [4].

Furthermore, it is worth noting that kinematic alignment may help to further minimize potential functional differences. Shelton et al. [30] reported that compared to a kinematically-aligned primary TKA, a failed UKA revised to a kinematically-aligned TKA led to comparable postoperative outcomes.

Overall postoperative patient satisfaction was also highest among UKA patients, followed by TKA patients, converted UKA patients, and then revised TKA patients. The differences, neither between primary UKA and primary TKA nor between converted UKA and primary TKA, arrived at the level of significance, whereas converted UKA patients were significantly less satisfied than their primary UKA counterparts. It is important to note that postoperative patient satisfaction can be highly dependent on preoperative patient expectations. In our experience, UKA patients are more likely to expect a “forgotten knee” than TKA ones, which would explain the lack of a significant difference in patient satisfaction despite a significant difference across all assessed outcome scores between primary UKA and TKA. The loss of the functional benefit of UKA after conversion to TKA would then explain the significant decrease in patient satisfaction without a significant difference in outcome scores between primary TKA and converted UKA. As a result, UKA revision should always be performed with consideration, regardless of its perceived ease. A breakdown by sex showed that overall patient satisfaction with primary arthroplasty tended to be higher in men than in women. Similar results were found by Munzinger et al. [31] who implanted TKA in 174 women and 86 men.

Patients receiving UKA, TKA, and converted UKA reported improved overall health status postoperatively. In contrast, most of the revision TKA patients reported a worsening of their general health status postoperatively. It’s worth mentioning that primary arthroplasty was perceived as significantly superior to revision arthroplasty. The difference between UKA and TKA as well as between converted UKA and revised TKA was not significant. Revision arthroplasty requires specialized anaesthesia, surgical experience, physiotherapy, and nursing care. These unique perioperative needs must be considered in light of differences in baseline characteristics and postoperative outcomes, which result in variations in recovery.

UKA patients tended to return to work and activities of daily living fastest, followed by TKA patients. As expected, revision TKA patients performed worst. The time required for patients to fully return to their private or professional life after surgery is an important factor, especially for younger, working patients, because the indirect costs, resulting from long postoperative reduced work performance, absence from work, or even resultant unemployment, can be enormous and thus an argument against the surgery [32]. Compared to the preoperative situation, UKA patients rated their overall ability to engage in sports as better, TKA patients as unchanged, and converted UKA and revision TKA patients as worse than before surgery. This is consistent with previous studies showing that the Oxford III UKA for medial knee osteoarthritis achieved a high level of patient satisfaction in terms of physical and sports activities without an increased risk of complications [33, 34].

This study has several limitations. First, it did not include information on postoperative (re-) revision rates, which were reportedly higher for converted UKA than for primary TKA [17, 28, 35]. While Sierra et al. (2013) and Lombardi et al. (2018) reported that the revision rate of failed UKA was comparable to the revision rate of primary TKA and significantly lower than the revision rate of failed TKA [10, 36], data from the New Zealand National Joint Registry showed that the revision rate of converted UKA was four times higher than that of primary TKA [19]. Data from the Australian Joint Registration Center showed that the revision rate of UKA converted to TKA was more than 2 times higher than that of primary TKA within 3 years after surgery, with aseptic loosening being the most common type of failure with an estimated rate of 46% [6, 37]. However, compared to the revision rates of failed TKA, those of converted UKA were reported to be lower [29, 37]. Second, all conversions of UKA in this study were performed without the use of revision implants, i.e., without augments and stems, which might have had a positive impact on the observed clinical outcome of the converted UKA. The use of autologous bone grafts by transferring a bone slice from the lateral to the medial proximal tibia has been shown to be a safe revision technique with good midterm results [38]. In general, as stated by Chou et al. (2012) and Wynn et al. (2012), tibial bone defects are considered to be the predominant challenge in the revision of UKA, and stems, augments, bone grafts or thicker inlays are often required to address this difficulty, leading to inferior clinical outcomes to those of primary TKA [39, 40]. Third, postoperative complications and the length of hospital stay were not considered in this study. Fourth, the inclusion criterion of identical standard implants in both the primary TKA and converted UKA group and the retrospective study design led to a selection bias as well as the impossibility of analyzing parameters other than those previously collected during clinical routine. The number of patients was limited due to the mentioned inclusion criterion, but it was comparable to those of former studies [11, 12, 15, 18]. The mean follow-up of 4 years allowed for the assessment of a stable postoperative situation.

## Conclusion

The results of the present study are of high clinical relevance. First, UKA led to better functional results than TKA. Second, if the identical standard implants were used in the revision scenario, converted UKA might achieve comparable clinical results and patient satisfaction as primary TKA. Third, revision TKA was associated with poorer functional results than primary arthroplasty. These findings may be helpful in counselling patients who are candidates for UKA. Finally, we believe that the foundation for the revision of failed UKA using standard implants has already been laid during primary implantation and should be considered at this stage.

## Data Availability

The datasets used and/or analyzed during the current study are available from the corresponding author on reasonable request.
